# Cerium Oxide Nanoparticles: A New Therapeutic Tool in Liver Diseases

**DOI:** 10.3390/antiox10050660

**Published:** 2021-04-24

**Authors:** Gregori Casals, Meritxell Perramón, Eudald Casals, Irene Portolés, Guillermo Fernández-Varo, Manuel Morales-Ruiz, Victor Puntes, Wladimiro Jiménez

**Affiliations:** 1 Service of Biochemistry and Molecular Genetics, Hospital Clinic Universitari, Centro de Investigación Biomédica en Red de Enfermedades Hepáticas y Digestivas (CIBERehd), Institut d’Investigacions, Biomèdiques August Pi i Sunyer (IDIBAPS), 08905 Barcelona, Spain; casals@clinic.cat (G.C.); mperramon@clinic.cat (M.P.); iportoles@clinic.cat (I.P.); guillermo.fernandez@ciberehd.org (G.F.-V.); morales@clinic.cat (M.M.-R.); 2 Comission for the Biochemical Assessment of Hepatic Disease-SEQCML, 08036 Barcelona, Spain; 3 School of Biotechnology and Health Sciences, Wuyi University, Jiangmen 529020, China; wyuchemecm@126.com; 4 Departament of Biomedicine, University of Barcelona, 08036 Barcelona, Spain; 5 Vall d’Hebron Research Institute (VHIR), 08035 Barcelona, Spain; victor.puntes@icn2.cat; 6 Institut Català de Nanociència i Nanotecnologia (ICN2), CSIC, The Barcelona Institute of Science and Technology (BIST), Campus UAB, Bellaterra, 08193 Barcelona, Spain; 7 Institució Catalana de Recerca i Estudis Avançats (ICREA), 08010 Barcelona, Spain

**Keywords:** nanoceria, liver steatosis, liver regeneration, hepatocellular carcinoma

## Abstract

Oxidative stress induced by the overproduction of free radicals or reactive oxygen species (ROS) has been considered as a key pathogenic mechanism contributing to the initiation and progression of injury in liver diseases. Consequently, during the last few years antioxidant substances, such as superoxide dismutase (SOD), resveratrol, colchicine, eugenol, and vitamins E and C have received increasing interest as potential therapeutic agents in chronic liver diseases. These substances have demonstrated their efficacy in equilibrating hepatic ROS metabolism and thereby improving liver functionality. However, many of these agents have not successfully passed the scrutiny of clinical trials for the prevention and treatment of various diseases, mainly due to their unspecificity and consequent uncontrolled side effects, since a minimal level of ROS is needed for normal functioning. Recently, cerium oxide nanoparticles (CeO_2_NPs) have emerged as a new powerful antioxidant agent with therapeutic properties in experimental liver disease. CeO_2_NPs have been reported to act as a ROS and reactive nitrogen species (RNS) scavenger and to have multi-enzyme mimetic activity, including SOD activity (deprotionation of superoxide anion into oxygen and hydrogen peroxide), catalase activity (conversion of hydrogen peroxide into oxygen and water), and peroxidase activity (reducing hydrogen peroxide into hydroxyl radicals). Consequently, the beneficial effects of CeO_2_NPs treatment have been reported in many different medical fields other than hepatology, including neurology, ophthalmology, cardiology, and oncology. Unlike other antioxidants, CeO_2_NPs are only active at pathogenic levels of ROS, being inert and innocuous in healthy cells. In the current article, we review the potential of CeO_2_NPs in several experimental models of liver disease and their safety as a therapeutic agent in humans as well.

## 1. Antioxidant Properties of CeO_2_NPs

The therapeutic ability of cerium oxide nanoparticles (CeO_2_NPs) to act as a redox buffer and balance redox homeostasis in conditions in which increased oxidative stress plays a pathogenic role makes them one of the most promising medical nanoparticles to address the different conditions related to chronic inflammation and oxidative stress. CeO_2_NPs are considered a multi-enzymatic nanozyme, since they can participate in numerous metabolic reactions mimicking the activity of endogenous enzymes. The catalytic capacities and other industrial activities of CeO_2_ have been known and applied for more than a century [1]. However, their powerful medical potential was not recognized until recently. The first report was from Beverly Rzigalinski and co-workers [2], who, with their “somewhat serendipitous discovery”, showed that CeO_2_NPs prolonged the lifespan of brain cell cultures for periods of up to 6–8 months [3,4]. In their works, CeO_2_NPs were intended to be used as a drug delivery vehicle. Unexpectedly, they realized that brain cells incubated with CeO_2_NPs were still alive and actively signaling “as robustly as freshly prepared cultures” after months in the incubator [3]. Afterwards, they started a new set of experiments to finally describe for the first time CeO_2_NPs of less than 20 nm in size prolonging the life of mixed brain cell cultures and neurons within these cultures for periods of up to 6–8 months. A patent was presented [5] and three seminal abstracts were made public in 2003 [6,7,8].

From this point on, the interest in CeO_2_NPs and their catalytic properties against the accumulation of free radicals rapidly grew and expanded to other medical areas. In 2005, the first report of CeO_2_NP protection against b radiation-induced damage appeared. In the work of Tarnuzzer et al. [9], CRL8798 cells (immortalized normal human breast epithelial cell line) and MCF-7 (breast carcinoma cell line) were exposed to radiation and further treated with CeO_2_NPs. The results showed that CeO_2_NPs conferred radioprotection to the normal human breast line but not to the tumoral line. In 2006, the first report on the use of CeO_2_ in neurology was published. Schubert et al. [10] found that CeO_2_NPs are neuroprotective and can limit the amount of ROS needed to decrease the viability of nerve cells using a HT22 hippocampal nerve cell line. The following year, Das et al. [11] showed similar neuroprotective effects using adult rat spinal cord neurons. Additionally, in 2006 the use of CeO_2_ in ophthalmology was reported for the first time. Chen et al. [12] showed how CeO_2_NPs can prevent retinal degeneration induced by intracellular peroxides, thus preserving retinal morphology and preventing loss of retinal function. These authors performed both in vitro and in vivo experiments. Retinal protection was shown for primary cells dissociated from rat retina incubated with CeO_2_NPs and through the injection of a suspension of CeO_2_NPs into the vitreous of both eyes in an albino rat light-damage model. The following year, the first report of the cardioprotective effects of CeO_2_NPs appeared. Niu et al. [13] showed that i.v.-injected CeO_2_NPs in a transgenic murine model of cardiomyopathy reduced myocardial oxidative stress and endoplasmic reticulum stress and suppressed the inflammatory process. The first report we have found on the use of CeO_2_NPs in the context of liver disease is the 2013 work of Hirst et al. [14]. These authors showed that CeO_2_NPs, administered both i.v. and intraperitoneally (i.p.) to mice with carbon tetrachloride (CCl_4_)-induced liver toxicity, showed similar and more sustained effects than mice treated with N-acetyl cysteine (NAC), a common therapeutic to reduce oxidative stress. Nowadays, many reports and studies are constantly appearing with very promising results [15]. 

The mechanisms responsible for these therapeutic activities are related to the CeO_2_NP dual status of oxidation. In nature, cerium oxide can form two main crystal structures, cerium (IV) oxide (CeO_2_) and cerium (III) oxide (Ce_2_O_3_), with CeO_2_ being the most stable phase at room temperature and under atmospheric conditions. When the size of the particle is reduced to the nanometric regime, a large amount of surface defects appear in the CeO_2_ crystal structure, primarily caused by the reversible removal of oxygen atoms from the surface. Electrons left behind by released oxygen localize on empty f states of cerium ions, being reduced from Ce^4+^ to Ce^3+^ [16]. Hence, CeO_2_NPs have two oxidation states, Ce^3+^ and Ce^4+^, which confer upon them the characteristic of generally two auto regenerative redox cycles between Ce^3+^ and Ce^4+^, which are associated with the release of oxygen at the surface. This translates into powerful antioxidant properties, since the lack of oxygen enables the appearance of reactive sites on the surface of the nanoparticles, and these reactive sites are able to scavenge free radicals [14]. 

It is therefore the ability of CeO_2_NPs to change their oxidation state depending on the surrounding environment and thereby be involved in oxidation reactions that makes CeO_2_NPs such a promising material for use in biomedicine. The key factor is their ability to participate in biochemical redox processes, especially in the modulation of oxidative stress in living organisms. Briefly, oxidative stress occurs when cells are no longer able to control the physiological levels of free radicals (molecules with unpaired electrons in the valence shell and that are therefore highly chemically reactive). The normal metabolism of the cell produces free radicals, principally reactive oxygen species (ROS), and control their levels. Under many conditions of stress (e.g., exposure to UV radiation, diet), the accumulation of ROS overwhelms defense mechanisms, resulting in damaged cellular structures. Furthermore, the network of endogenous antioxidants (superoxide dismutase [SOD], catalase, peroxidase, glutathione (GSH), etc.) is complex in itself and is interrelated [17] (for instance, SOD produces H_2_O_2_ as a product of the degradation of O_2_, etc.). The accumulation of ROS is known to lead to DNA damage (by the oxidation of nucleotides and induction of mutagenesis), protein degradation, and lipid peroxidation, which are reactions that ultimately lead to inflammatory processes [18]. In turn, inflammation itself triggers a higher ROS production by the cells of the immune system as a mechanism of innate defense to form a less friendly environment for pathogens [19]. Therefore, an excess of ROS induces inflammation. However, the reverse sequence of these events is also true; inflammation induces ROS to alter immune cell phenotypes and activate them in a type of positive reciprocal feedback loop [20,21,22,23,24].

To decrease the levels of ROS and stop this vicious ROS inflammation cycle, the oxygen electronic state of these ROS is changed and they are converted into benign molecules such as O_2_ and H_2_O through reactions catalyzed by different endogenous enzymes. The redox activity of CeO_2_NPs is similar to that of biological antioxidants, and during the last two decades the following have been described for CeO_2_NPs: SOD activity [25,26] (the conversion of superoxide anion into hydrogen peroxide and finally oxygen), catalase activity [27,28] (hydrogen peroxide into oxygen and water), and peroxidase activity [29] (hydrogen peroxide into hydroxyl radicals), as well as nitric oxide (NO) scavenging ability [30], among others. Here, it is worth noting that thanks to this auto-regenerative redox cycle, CeO_2_NPs can participate in these enzymatic reactions by catalytically degrading the excess of different ROS—i.e., without being totally consumed in the reaction and enabling longer and sustained activity compared with the shorter half-life of classic (small molecule) antioxidants. In addition, when doing this, due to their peculiar electronic structure, CeO_2_NPs act as a buffer, balancing redox homeostasis. This means that their biological activity is only carried out in the case of ROS overproduction [31] and they become a rather inert material in healthy physiological conditions, slowly dissolving into innocuous cerium ions which are finally expulsed via the urinary track or the hepatic route [32,33]. Thus, CeO_2_NPs are considered one of the major players in what has recently been called “nanocatalytic medicine” [34], or “ROS-based nanomedicine” [35], understood as the use of nanozymes [36,37] and antioxidant nanomaterials [38] (and also photocatalysts and electrocatalysts) to initiate catalytic (enzymatic) reactions and modulate biological microenvironments to generate therapeutic effects. 

Thus, over the last few years the many advantages of CeO_2_NPs over classic (small molecule) antioxidants have been described. Classic antioxidants, such as SOD, ascorbic acid, resveratrol, colchicine, eugenol, and vitamin E, have shown only limited success in clinical applications [1,39,40]. This has been called the antioxidant paradox, since they were thought to have a higher bioactivity against oxidative stress and ageing. The shortcomings of these antioxidants accounting for the unsuccessful clinical trials are their limited activity, since they are metabolized—i.e., after the reaction they become inactivated (hence, they are considered to have a short half-life)—and the fact that they often scavenge only one free radical [23]. This limited activity is also related to the reaction environment. For instance, while vitamin C acts in intracellular and extracellular environments, vitamin E acts in the membrane. In addition, to date the state of the design of efficient small-molecule antioxidants with targeted activity is still somewhat lacking. CeO_2_, in its nanoparticle form, can overcome these drawbacks and may provide to the field of medicine an effective long-lasting antioxidant for the treatment of a broad spectrum of diseases associated with free radical production, especially in the cases of autoimmune diseases, chronic inflammation, organ rejection, pathogenic immune response, and conditions related to aging. First, because NPs can be easily functionalized by targeting peptides or molecules, they can thus be designed to be used for local targeting and delivery to specific cellular types or damaged tissues. In addition, as mentioned previously, CeO_2_NPs scavenge different ROS without being consumed during the reaction. Thus, even at low doses, they can be more effective and have long-lasting activity in a multiplicity of cross reactions between ROS and inflammation at any level, which will ultimately allow disconnecting these two events [15].

## 2. Liver Regeneration

The liver has the capacity to regenerate owing to the ability of adult hepatocytes to proliferate upon toxic injury and infection. Liver regeneration occurs similarly in humans and rodents. In 1931, Higgins and Anderson established the first experimental model of liver regeneration in rodents named partial hepatectomy (PHx). By means of this procedure and benefiting from the multilobular structure of rodent liver, it is possible to remove two thirds of the liver by the resection of the median lobe and the left lateral lobe [41]. Thenceforth, this model has been widely used to study liver regeneration, liver function, and response to trauma and stress.

Liver regeneration is a compensatory process driven to restore liver function. After PHx, the remnant liver enlarges until the original liver mass is reestablished because of the cellular hyperplasia of mature liver cells. Typically, 7 days after PHx the liver restores the majority of its mass and after 3 weeks complete restoration is accomplished [42,43]. 

The process of liver regeneration after PHx is complex and implicates the coordination of many initiation and termination pathways. Although hepatocytes are the major contributors to liver regeneration, it is not solely these cells that participate in this process; cholangiocytes, hepatic stellate cells (HSCs), Kupffer cells, and liver sinusoidal endothelial cells are also involved. 

Liver regeneration can be explained by three different phases. The early activation phase triggers a signaling cascade of cytokines that activate the transcription factors needed for liver regeneration. During the second phase, DNA synthesis and cell proliferation take place due to mitosis. In the termination phase, the original liver mass is restored by hyperplasia and liver regeneration stops [44].

Early activation phase: In this initial phase, different cytokines and growth factors are responsible for the transition between quiescence and mitosis, allowing hepatocytes to enter in the G1 phase of the cell cycle. Kupffer cells rapidly regulate the early stage of regeneration by secreting interleukin 6 (IL-6) and tumor necrosis factor alpha (TNFα) [42]. IL-6 is essential for the proper functioning of the liver, since it participates in acute phase response and mitosis. IL-6 is a key mediator of gene expression activation during liver regeneration, triggering the expression of 36% of genes that activate in the early phase of this process. Hepatic macrophages and hepatocytes produce IL-6 rapidly after PHx. The linkage of IL-6 with its receptor (IL-6R) triggers a signaling cascade that induces the activation of transcription of certain genes via STAT3 phosphorylation and activates mitogen-activated protein kinase (MAPK), which initiates mitosis, via gp130 phosphorylation [42,43]. After PHx, Kupffer cells rapidly express TNFα, which up-regulates the nuclear factor kappa-light-chain enhancer of activated β cells (NF-κB) expression through the activation of IL-6 transcription. TNFα can act as both a protector and promoter of liver injury [42,45,46]. Nonetheless, NF-κB signaling mediated by TNFα is anti-apoptotic; mice deficient in TNF receptor 1 or 2 presented a delayed regeneration and lower activation of NF-κB [47,48]. In this phase, the action of growth factors that stimulate cellular replication is also fundamental. As such, hepatocyte growth factor (HGF) is implicated in promoting hepatocyte proliferation and inducing DNA synthesis. Immediately after PHx, the activation of the urokinase-type plasminogen activator (uPA) occurs, allowing the conversion of plasminogen into plasmin, and, consequently, activating metalloproteinases [49]. Thereafter, there is a remodeling of some components of the extracellular matrix (ECM). When the vascular endothelial growth factor (VEGF) binds to endothelial cells, HSCs release the inactive HGF precursor [42]. uPA mediates the activation of the HGF precursor by the cleavage and release of HGF. HGF binds to the Met receptor on hepatocytes, activating the PI3K, AKT, and S6 kinase signal-transduction pathways (TOR). Upon HGF signaling, transforming growth factor alpha (TGFα) is released, triggering a cascade of different downstream signals that together activate TOR. Upon uPA blockade, HGF action is delayed, hindering liver regeneration [50]. In brief, HGF and TGFα are the main regulators of the mitogenic response of the liver. When liver regeneration terminates, the normal state of ECM is reestablished. 

Proliferation phase: in the normal liver, functional hepatocytes are found in a quiescent state, maintaining their ability to divide in response to damage or infection. After PHx, hepatocytes are the first cells to undergo cell division; they also act as activators of proliferation of other hepatic cell types by producing mitogenic signals. In mice, the peak of hepatocyte proliferation occurs at between 36 and 48 h, with the highest DNA synthesis being found at 40 h; in rats, the peak is observed at 24 h [42,51,52].

Termination phase: The mechanisms leading to the termination of regeneration have still not been completely elucidated. The speed of the hepatic regenerative process is determined by the mass amount of the excised liver, which is proportionally correlated. At between 40% and 70% liver resection, the process evolves at an optimal speed. If only 30% or less of the liver is removed, the speed notably decreases and the growth of the remnant liver slows down, even if the mass is ultimately restored. The resection of more than the 85% of the liver volume is associated with mortality and poor regeneration. However, when the original liver mass is restored, liver regeneration completely stops [53]. Cytokines and growth factors are also implicated in the termination phase of this process, regulating liver size. Through the action of Janus kinases (JAK), the suppressor of cytokine signaling-3 (SOCS-3) impedes the phosphorylation of STAT-3, preventing and blocking cytokine signaling. IL-6 is the main regulator of the mRNA expression levels of SOCS-3. After PHx, SOCS-3 is up-regulated, leading to the down-regulation of STAT-3, and, consequently, down-regulating IL-6 in a negative feedback manner [42,54]. The duality of IL-6 acting as a proliferative and apoptotic agent might explain why IL-6 over-expression impedes cell growth, hindering liver regeneration [55]. TGFβ is the most well-known anti-proliferative factor in hepatocytes. HSC produces TGFβ and is over-expressed during liver regeneration. However, it has been described that hepatocytes become resistant to TGFβ. Within 48 h after PHx, hepatocytes decrease the expression of TGFβ receptors and are then able to proliferate during regeneration, despite the high levels of TGFβ [51,56].

Smad proteins are intracellular effectors of TGFβ signaling. These proteins become active through interaction with different receptors, consequently translocating into the cell nuclei where they activate gene transcription. Smad proteins are slightly activated in quiescent hepatocytes, and their activation increases in liver regeneration. During regeneration, the expression of the inhibitors of the TGFβ/Smad pathway, SnoN and Ski, increases. These inhibitors impede transcription and can favor cellular resistance to TGFβ through their union to SMAD proteins [57]. 

### Cerium Oxide Nanoparticles as a Driver for Liver Regeneration

To maintain optimal physiological functions and structural integrity, it is essential to ensure redox homeostasis. A proper balance between oxidants and antioxidants is obtained by controlling the production of ROS and RNS. ROS are oxygen free radicals, such as superoxide, with the addition of non-radicals, such as hydrogen peroxide, which are generated during the metabolism process of oxygen. RNS, such as nitrogen dioxide or NO radicals, are derived forms of NO and superoxide that arise from the action of inducible nitric oxide synthase (iNOS) and nicotinamide adenine dinucleotide phosphate (NADPH) [58,59]. 

Under physiological conditions, the oxidation of molecules resulting from the breakage of the DNA strand induced by ROS/RNS is normally held at bay, since the cellular production of anti-oxidants acts as a barrier defense. Amongst them, we find enzymatic anti-oxidants such as SOD, catalase, and glutathione peroxidase, as well as non-enzymatic anti-oxidants, such as vitamin-E, GSH, beta-carotene, tocopherol, and ascorbate [60,61,62]. 

ROS production is a natural process derived from the aerobic metabolic pathways, and under physiological conditions these molecules exert beneficial roles. For instance, ROS serve as a defense against microorganisms; they modulate gene expression in response to growth factors, hormones, cytokines, and extracellular ATP [60,63]. However, an imbalance in the production or elimination of ROS or a decreased availability of antioxidants leads to the commonly known state of oxidative stress. In general, a sustained situation of oxidative stress may induce cell death, causing tissue damage. 

Due to its highly metabolic functions, the liver is very sensitive to redox imbalances. Proteins, lipids, and DNA in hepatocytes are the molecules that are mainly affected by oxidative stress. Cysteine, tyrosine, tryptophan, and histidine are the main amino acids that are compromised by high levels of ROS [60]. Proteins rich in these amino acids are direct targets of ROS, becoming modified and consequently proteolyzed after their action. 

Imbalances in ROS levels that lead to oxidative stress are crucial in liver diseases and chronic liver injury. Oxidative stress causes hepatic damage by altering proteins, lipids, and DNA, as well as modulating pathways involved in gene transcription, protein expression, cell apoptosis, and HSC activation. In the pathological setting of the liver, oxidative stress exacerbates fibrosis by activating HSCs, steatosis by causing lipid peroxidation, and inflammation that can lead to chronic hepatitis by triggering mitochondrial dysfunction and immune cell infiltration. All these pathological conditions can contribute to the development of hepatocellular carcinoma (HCC) [59]. Despite ROS playing an important role in liver disease, a therapeutic approach directly targeting ROS is still not available in the clinical setting. 

In recent times, CeO_2_NPs have been tested for biomedical purposes, since their ability to scavenge free radicals may serve as a new therapeutic tool to treat oxidative stress-related diseases. In this regard, the beneficial effect of CeO_2_NPs in liver pathologies has recently been described in several studies. However, despite the increasing interest in the hepatoprotective properties of nanoceria in liver diseases, little is known about their role in liver regeneration. As mentioned above, during liver regeneration it is essential to maintain the proliferative state until the original liver mass is restored. However, in many liver diseases the accumulation of ROS may impede optimal regeneration due to the induction of apoptosis because of lipid peroxidation, subsequently preventing the resolution of tissue damage. ROS act as mediators in the regulation of different growth factors, transcription factors, and cell cycle proteins such as β-catenin, cyclin D, p53, and NF-E2-related factor 2 (Nrf2) [64,65,66,67,68]. All these proteins are essential for the regenerative process, and improper regulation results in detrimental effects on liver regeneration. 

The transcription factor Nrf2 is a pivotal agent in the protection against oxidative stress. Nrf2 is involved in the regulation of the expression of antioxidants, such as glutathione-S-transferase (GST), glutamate-cysteine ligase catalytic subunit (GCLC), and NADPH quinine oxidoreductase 1 (NQO1) [69]. Nrf2 binds to a specific site in the promoter region of its target genes named antioxidant response element (ARE) [70]. Beyer et al. further described the role of Nrf2 in liver regeneration in relation to ROS production. In their Nrf2 knockout (KO) mice, they studied liver regeneration upon PHx and found a significant delay in regeneration in the absence of Nrf2 as well as enhanced hepatocyte apoptosis. In this study, they also observed reduced GST activity in the KO mice and increased oxidative stress [71]. Later on, the role of Nrf2 as an activator of augmenter of liver regeneration (ALR) was described [72]. Therefore, it was concluded that Nrf2 is a key regulator of the redox state. From these results, the relationship between the redox state and liver regeneration is evident. Francés et al. analyzed the effects of free radical scavengers in the early stages of PHx. It is known that diabetes mellitus induces lipid peroxidation through the generation of hydroxyl radicals. For this reason, they used a streptomycin-induced diabetes model in rats and subjected them to treatment with desferoxamine (DES) or tempol (TEM), two known free radical scavengers, and studied their effects over 24 h after hepatectomy. Their results show a decreased ROS production and the activation of caspase-3 upon DES and TEM treatment, thereby preventing apoptosis and ameliorating liver regeneration in a diabetic setting [73].

**Figure 1:**
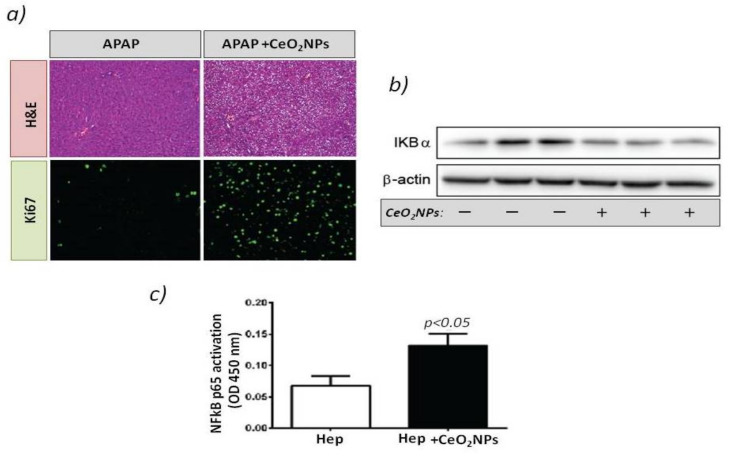
(**a**) Rats received vehicle or 1 g/kg acetaminophen (APAP) after CeO_2_NP treatment. The top panels show hematoxylin-eosin stained liver sections. The bottom panels show immunostaining for Ki-67 (green) used as a marker of hepatocellular proliferation. Magnification: 100×. (**b**) Western blot for IκβBα expression in the HepG2cell line treated with vehicle or CeO_2_NP. (**c**) Transcription factor immunosorbent assay for p65 activity in the cell line HepG2 treated with vehicle or CeO_2_NP (adapted from reference [68]).

It was not until 2019 that Cordoba-Jover et al. [68] first studied the effects of using CeO2NPs on liver regeneration using the experimental model of PHx and acetaminophen (APAP)-induced liver injury in rats. In the context of PHx, rats were administered with CeO_2_NPs or vehicle two weeks before PHx and sacrificed 6 days after surgery. Rats treated with CeO_2_NPs exhibited significantly increased liver regeneration and hepatocyte proliferation compared to control groups [68]. In the context of APAP-induced liver injury, the therapeutic effect of CeO_2_NPs was compared with NAC, the clinical gold-standard treatment. CeO_2_NPs and NAC treatment decreased early liver damage in hepatic tissue after APAP overdose. However, only the effect of CeO2NPs was associated with a significant increment in hepatocellular proliferation. In addition, treatment with CeO_2_NPs increases transcription factor NF-kB activation by decreasing the IKBα expression (Figure 1). The link between CeO_2_NP activity and the downregulation of IKBα seems to lie in the inhibition of the IkB kinase (IKK) complex by high levels of oxidative stress. In the absence of oxidative stress, IKK phosphorylates IkB proteins [74], leading to protein ubiquitination, which is followed by the proteasome-mediated degradation of IkB proteins.

These results agree with studies that showed that NF-kB activity is needed for liver regeneration and that impaired NF-kB activation is associated with embryonic lethality and liver degeneration. This study reflects the beneficial properties of CeO_2_NPs and the positive impact on stimulating liver regeneration.

## 3. Fatty Liver Disease

The definitions of non-alcoholic fatty liver disease (NAFLD) have been based on the presence of fat accumulation (steatosis) in hepatocytes in the absence of significant alcohol consumption or other known causes of liver disease [75,76]. A more recent definition proposed by a panel of international experts considers metabolic dysfunction-associated fatty liver disease (MAFLD) to be a more appropriate term for this liver disease, which is highly associated with known metabolic dysfunctions [77]. Regardless of alcohol consumption or other concomitant liver diseases, the new diagnosis of MAFLD is based on the evidence of hepatic steatosis in addition to one of the following three criteria—namely, (1) overweight/obesity, (2) type 2 diabetes mellitus, or (3) two additional metabolic risk abnormalities [77]. 

MAFLD is currently the most common cause of liver disease. It already affects one quarter of the adult population [78] and is a major health and economic burden [79]. It is associated with increased cardiovascular and liver-related morbidity and mortality and, at present, there is a lack of approved pharmacotherapy [77]. Therefore, the identification of new therapeutic strategies is urgent in order to reduce the increase in chronic liver disease that can be derived from the high prevalence of MAFLD among the population.

### 3.1. Lipid Peroxidation

Two main histological categories may be considered in MAFLD: simple fatty liver, with a favorable clinical outcome, and non-alcoholic steatohepatitis (NASH), characterized by inflammation in addition to the fat infiltration of the liver, and at higher risk of developing fibrosis, cirrhosis, liver failure, and hepatocarcinoma. Fat accumulation in the hepatocytes is the result of an increased inflow of free fatty acids, de novo lipogenesis, or impaired fat oxidation. Elevated hepatic oxidative stress and lipid peroxidation play roles in the pathogenesis of MAFLD and NASH. Increased ROS generation triggers lipid peroxidation, the release of inflammatory cytokines, and cell death. Both biologically active lipid peroxidation products and cytokines act together to trigger the diverse hepatic lesions of NASH by inducing hepatic inflammation and fibrosis, which eventually lead to end-stage liver disease. Patients with NASH display both an increase in ROS and nitrogen species production and a lack of endogenous antioxidant defenses [78]. ROS can attack polyunsaturated fatty acids and initiate lipid peroxidation within the cell, which results in the formation of aldehyde by-products such as malondialdehyde (MDA) and 4-hydroxynonenal. These by-products, with longer half-lives than ROS and easily diffusible, amplify the effects of oxidative stress [79].

MDA, which results from the lipid peroxidation of polyunsaturated fatty acids, is the major lipid oxidation product in biological samples. Therefore, MDA and related thiobarbituric acid reactive substances (TBARS) are widely used as markers of lipid peroxidation. At a dose of 50 μg/mL, CeO_2_NPs (25 nm) decreased cell viability and increased the production of ROS and MDA in HCC SMMC-7721 cells cultured in basal conditions [80]. This effect was not observed at a dose <50 μg/mL, suggesting that very high doses of CeO_2_NPs may induce oxidative stress in control cells. In contrast, protective effects of CeO_2_NPs against lipid peroxidation have been found under different experimental conditions of liver disease. Thus, HepG2 cells incubated in high-glucose medium showed an increase in ROS formation, as well as TBARS levels that were remarkably reduced after treatment with 50 nM (8.5 μg/mL) nanoceria [81]. In vivo evidence of a potential effect of CeO_2_NPs in reducing lipid peroxidation includes the study of Hirst et al. [14] in a BALB/c mice model of liver disease induced with CCl_4_. Treatment with CeO_2_NPs (4 nm) reduced MDA in plasma after 2 weeks of CCl_4_ administration. In another study, the i.p. administration of CeO_2_NPs to mice with D-galactoseamine and lipopolysaccharide-induced hepatotoxicity resulted in decreased levels of TBARS in comparison with non-treated animals [82]. Additionally, a reduction in MDA levels was observed after the administration of CeO_2_NPs (25 nm) to Sprague Dawley rats with hepatic toxicity induced by doxorubicin [83] and to Wistar rats with fatty liver induced by a methionine- and choline-deficient diet [84]. A decrease in lipid peroxidation was also observed in Wistar rats with monosodium glutamate-induced obesity when treated orally with CeO_2_NPs in two-week courses alternated with two-week breaks for 3 months. In comparison to non-treated rats, rats receiving CeO_2_NPs presented a reduced liver tissue content of diene conjugates, TBA-active products, and Schiff bases [85]. The oral administration of CeO_2_NPs (<25 nm) also protected albino rats against hepatotoxicity induced by fipronil. The effects included reduction in the hepatic levels of MDA and nitric oxide, and also an improvement in the hepatic activities of glutathione peroxidase and superoxide dismutase [86]. 

### 3.2. Liver Steatosis 

The effects of CeO_2_NPs on liver steatosis have also been evaluated. Kitchin et al. found significant effects on lipids in metabolomic studies evaluating the potential hepatotoxicity of CeO_2_NPs in human liver HepG2 cells [87]. Specifically, HepG2 cells were exposed for 3 days to two commercial CeO_2_ nanomaterials (8 and 58 nm) at 3 or 30 µg/mL. Significant increases in lipid metabolites after treatment with CeO_2_ nanopowders were observed and found to be almost exclusively related to the smaller size 8 nm CeO_2_ nanomaterial. Thus, cells treated with 8 nm CeO_2_NPs at 3 µg/mL increased the levels of 11 of 24 fatty acids around 1.3–1.5 fold, and cells incubated with the same nanoparticles at the higher dose of 30 µg/mL increased the levels of 20 of 24 fatty acids around 1.5–2 fold. In contrast, the effects of 58 nm CeO_2_NPs on lipids were minimal and only an increase of one fatty acid (1.4-fold) was found at the higher incubation dose of 30 µg/mL. In agreement with these results, fatty acid synthase (FASN) gene expression was upregulated (1.6 fold) only in HepG2 treated with 8 nm at the higher dose [88]. The same group further evaluated metabolomic effects on HepG2 cells after 3 days of exposure to CeO_2_NPs using different commercial CeO_2_ nanopowders and observed similar nanomaterial-induced elevations in fatty acids and monoacylglycerols [89]. In contrast, when HepG2 cells were exposed to oleic and palmitic acid to establish an in vitro model of hepatocellular steatosis, a significant reduction in the content of saturated fatty acids was observed in response to a treatment with colloidally stable (synthesized and stabilized with tetramethylammoniun hydroxide) 4 nm CeO_2_NPS (10 µg/mL) for 24 h. [90]. These contradictory results under normal and steatotic conditions may be related to the activity of CeO_2_NPs as nanozymes. Due to its particular electronic structure, CeO_2_ acts as a redox buffer—i.e., it balances redox homeostasis. Hence, its biological activity is mainly carried out in cases of an excess of ROS, while it is a rather inert material under physiological conditions [15]. In addition, the different doses employed and the aggregation of the commercial CeO_2_ nanopowders may have also an impact on the observed biological results. It is known that nanomaterials of dry origin are more unstable than synthesized colloidal stable NPs. In the mentioned works which used commercial CeO_2_ nanopowders [87,88,89], the particles needed to be resuspended in cell culture media prior to their incubation with cells. As expected, the characterization of the NPS in physiological media showed the presence of aggregates. Hence, it may be considered that these are different materials from those produced by wet chemistry routes in the laboratory, where the colloidally stable NPs are isolated and well dispersed in cell culture medium supplemented with serum. CeO_2_ colloids. This proneness of nanomaterials from dry origin to form aggregates in physiological media has been consistently associated with deleterious and toxic effects, as reported in a recent review [91].

**Figure 2:**
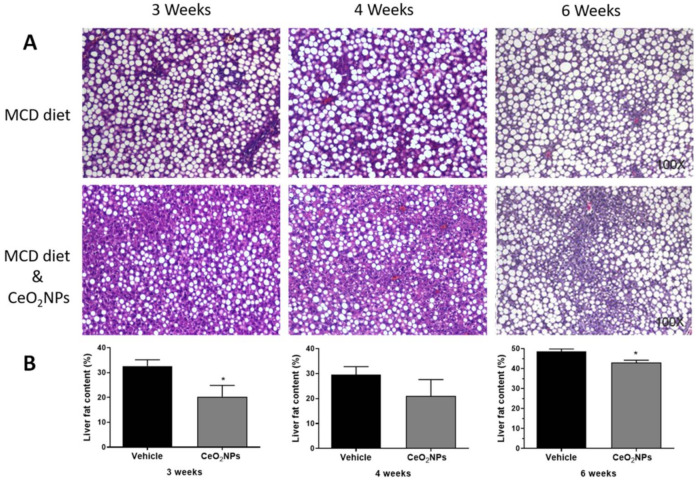
Effect of CeO_2_NPs on liver steatosis. (**A**) Hematoxylin and eosin representative liver sections obtained from methionine- and choline-deficient (MCD) diet Wistar rats receiving vehicle (MCD diet) or treated with CeO_2_NPs (MCD diet and CeO_2_NPs) for 3, 4, or 6 weeks. Original magnification 100x. (**B**) Quantitative measurements of liver fat content (%) in MCD diet rats receiving vehicle or treated with CeO_2_NPs. * *p* < 0.05 compared to MCD diet rats receiving vehicle. Unpaired Student’s *t* test (adapted from reference [84]).

There is some in vivo evidence in experimental models of liver disease reporting a reduction in liver steatosis with CeO_2_NPs treatment. Oró et al. [32] evaluated the systemic and hepatic effects of CeO_2_NPs (4 nm) in rats with liver fibrosis induced by CCl_4_. Eight weeks after the i.v. administration of CeO_2_NPs (0.1 mg/kg bw, twice weekly for two weeks), nanoparticles were mainly located in the liver, and a morphometric measurement of fat revealed an almost 50% reduction in total steatosis, which was associated with an amelioration of systemic inflammatory biomarkers and improved portal pressure, among other protective effects. Carvajal et al. [85] evaluated the effect of CeO_2_NPs (4 nm) in a rat model of NASH induced by a 6-week methionine- and choline-deficient diet. Rats were treated with CeO_2_NPs (i.v. 0.1 mg/kg bw) twice weekly during weeks three and four of the diet. Treatment with CeO_2_NPs reduced the size and content of hepatocyte lipid droplets, as assessed by histological morphometric measurement. This was associated with a reduction in the hepatic content of triglyceride- and cholesterol ester-derived fatty acids, as assessed by mass spectrometry analysis. These antisteatoic effects on the liver were also accompanied by a reduction in the hepatic levels of MDA and different inflammatory factors. In addition, unpublished results from our laboratory suggest similar or greater antisteatotic effects of CeO_2_NPs in the liver of rats fed with a methionine- and choline-deficient diet for 3 or 4 weeks, and, therefore, with a less established NASH, as observed by morphometric measurements of steatosis (Figure 2). 

Although a methionine- and choline- deficient diet is a classical dietary model of NASH suitable for assessing the hepatic effects of CeO_2_NPs, this model does not present the systemic metabolic abnormalities related to MAFLD. There are, however, few studies evaluating the effects of CeO_2_NPs in fatty liver associated with obesity models. Kobyliak et al. [92] studied the effects of CeO_2_NPs in a rat model of obesity induced by a neonatal injection of MSG that develops liver steatosis. CeO_2_NPs were administered orally (1 mg/kg bw) from one month of age in two two-week courses for 3 months. Histological examination of the liver at 4 months of age showed a reduction in hepatic steatosis and lobular inflammation in the CeO_2_NP-treated rats. Body weight, total liver lipids, and triglycerides were also significantly decreased. Rocca et al. [93] evaluated the anti-obesity potential of CeO_2_NPs, administrating them to 10-week-old normal Wistar rats twice a week for six weeks through i.p. injection at a dose of 0.5 mg/kg. Treated rats presented a lower body weight and reduced circulating levels of insulin, leptin, glucose, and triglycerides. In comparison, recent data from our lab [94] found lower circulating triglyceride levels in 14-week-old obese Zucker rats treated with CeO_2_NPS (0.1 mg/kg twice weekly in weeks 8 and 9; 4 nm) but did not find significant effects on the body weight or circulating levels of insulin and glucose. In addition, no significant effects of CeO_2_NPs on liver fat accumulation were observed by hepatic oil red staining or lipidomic analysis. Differences in the intrinsic characteristics of the nanomaterial such as size and surface states, along with their dose and route of administration, may be the basis of the discrepancy in the results between normal Wistar rats and obese Zucker rats. Importantly here, the evolution of CeO_2_NPs in physiological media in terms of protein corona formation and potential aggregation and/or corrosion depends on the extrinsic properties of the nanomaterials, which, in turn, depend on the characteristics on the media in which they are dispersed. Hence, different nanoparticle evolution and, thus, different biological impacts have been often observed for nanoparticles administered through different routes [15].

To summarize, lifestyle modifications (healthy diet and physical activity) are effective in the treatment of NAFLD. However, the long-term compliance is low and, therefore, several pharmacological treatments have been proposed, although none has shown significant efficacy or long-term safety sufficient to be recommended in clinical guidelines, with the exception of vitamin E and pioglitazone, which may be considered in some patients with NASH [75,76]. The evidence shown here points to a significant therapeutic potential of CeO_2_NPs in MAFLD, with significant effects on lipid peroxidation and liver steatosis in different experimental conditions. In view of future clinical applications, it is important to note that vitamin E, which can be considered as a main representative of “classical” antioxidants, is recognized as the drug with the most profound antisteatohepatitic effects [95]. However, the lack of efficacy in reducing hepatic fibrosis [96] limits their clinical value. Therefore, progress in the synthesis and design of a new generation of nanoctalysts such as antioxidant-based CeO_2_ nanomaterials should be aimed at overcoming the limitations of classical antioxidants in MAFLD. In fact, in contrast to classical antioxidants, which have short and no targeted activity, nanocatalysts such as CeO_2_NPs already present significant advantages that include their long residence time in tissues and their property of not being consumed during the reaction. However, advances in controlled biodistribution, functionalization, and/or combination in a single nanostructure with complementary agents targeting several activities and biological processes may be necessary to achieve the desired complete therapeutic effect in MAFLD. 

## 4. Liver Inflammation

### 4.1. Inflammation in the Development of Liver Diseases

Hepatic inflammation and sustained oxidative stress originate in response to a wide array of insults and are considered as common triggers of liver disease [97]. Following liver injury, damaged hepatocytes release a plethora of mediators such as growth factors, matrix metalloproteinases, and chemokines that promote the infiltration of immune cells and activate the apoptosis and regeneration of injured parenchymal cells [98]. HSCs also transdifferentiate into myofibroblast-like cells and migrate to sites of injury to secrete limited ECM. 

Intracellular self-structures named damage-associated molecular patterns (DAMPs), including mitochondrial components, adenosine triphosphate (ATP), nuclear proteins, and nucleic acids, are also released in the extracellular space during injury. They are recognized by pattern recognition receptors present on immune cells such as Kupffer cells, neutrophils, and dendritic cells [99,100]. In response, these cells are activated and induce the transcription of signaling pathways such as nuclear factor (NF)-κB, orchestrating a pro-survival and pro-inflammatory response positively modulating the expression of chemoattractant and proinflammatory mediators, including interleukin 1 alpha (IL-1α), IL-6, and TNF-α [101]. At the same time, these agents induce the expression of adhesion molecules in the site of injury to facilitate the recruitment of more either innate or adaptive immune cells and further stimulate them, thus establishing a highly hepatotoxic feedforward cycle [102]. 

**Figure 3:**
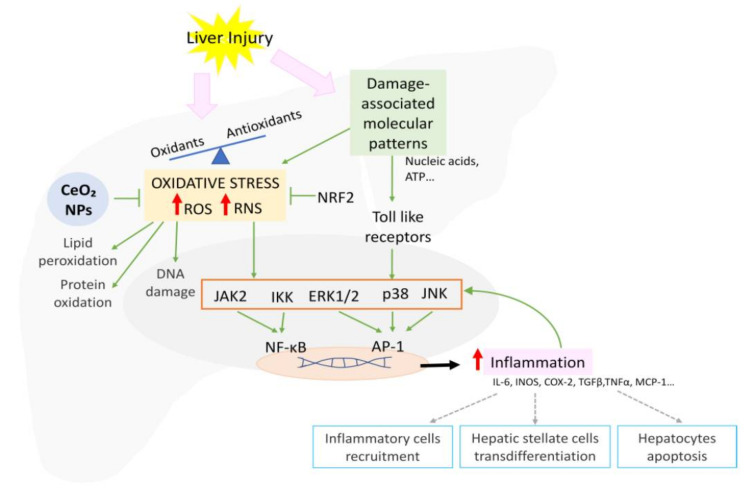
Schematic representation of the signaling pathways involved in oxidative stress-mediated inflammation and CeO_2_NP effects.

The overproduction of ROS and RNS during injury exceeding the buffer capacity of the cell results in mitochondrial dysfunction and DNA, lipid, and protein damage. Oxidative stress also activates signaling pathways including NF-κB, p38, ERK1/ERK2, JNK, and JAK, increasing proinflammatory gene transcription (Figure 3) [103]. In order to protect cells against injury, the elevated oxidative stress also induces the activation of the ARE, with Nrf being the master regulator [104]. Nrf2 modulates the expression of a myriad of genes such as SOD and glutathione reductase (GR), ultimately reducing the oxidative stress, cellular death, and inflammation [103]. 

When inflammation becomes chronic, there is a massive loss of the hepatic parenchyma; regeneration and protective pathways eventually fail; and huge quantities of ECM are secreted, leading to tissue fibrosis. Hepatic fibrosis can then further progress to cirrhosis and eventually lead to HCC [105]. Dysregulated inflammatory responses have also been associated, for instance, to hepatitis infections, alcoholic fatty liver disease, NAFLD, and ischemia/reperfusion (IR) injury [102]. 

### 4.2. Cerium Oxide Nanoparticles and Hepatic Inflammation

There is still a clinical need to develop more effective and safer therapies for most patients with liver diseases. Targeting pro-oxidant and inflammatory pathways could be a promising way to approach them, since oxidative stress has a central role in the progression of inflammation. Therefore, antioxidants are expected to interfere with proinflammatory signaling activation and subsequent tissue damage and death (Figure 3). Experimental evidence suggests that this is the case with CeO_2_NPs, which have the potential to attenuate hepatic inflammation regardless of the stage of liver disease [32,106]. 

Hirst et al. [14] reported that pretreating murine macrophages with nanoceria J774A.1 decreased ROS production as well as messenger RNA and protein levels of iNOS [105]. They also showed that macrophages stimulated with LPS and interferon γ and incubated with CeO_2_NPs diminished nitrate production in comparison to non-treated cells. Finally, although no differences were found in oxidative damage to DNA in mice with liver-induced toxicity by the intraperitoneal injection of CCl_4_ and treated with CeO_2_NPs, animals receiving the nanoparticles showed a greater reduction in lipid peroxidation compared to N-acetyl cysteine-treated animals [14]. 

In experimental rat liver fibrosis, nanoceria markedly reduced hepatic macrophage infiltration, oxidative-mediated endoplasmic reticulum stress messengers (Hspa5, Atf3), and the expression of M1-related genes (Il-1β, Tnf-α, iNos, and cyclooxygenase 2 (Cox-2)) in comparison to vehicle-treated animals [32]. Additionally, immortalized endothelial cells from the portal vein of cirrhotic rats (CH-iPVEC) treated with CeO2NPs presented decreased Il-6 expression. Furthermore, the secretome of these CH-iPVEC induced macrophage polarization from M1 to M2. Along this line, cirrhotic rats treated with nanoceria presented downregulated Il-6 in the portal vein [107]. In order to demonstrate whether the therapeutic properties of these NPs could be translated to human cells, a human-derived hepatocyte cell line named HepG2 was exposed to LPS and H_2_O_2_. CeO_2_NPs reduced ROS production and modified the messenger expression of proinflammatory and oxidative stress-related genes, including iNOS, myeloperoxidase (MPO), prostaglandin-endoperoxide synthase 1 (PTGS1), and neutrophil cytosol factor 2 (Ncf2) [108]. CeO_2_NPs were also demonstrated to be powerful anti-inflammatory agents in experimental NAFLD. Their administration in rats with MSG-induced obesity resulted in a lower serum amount of IL–12 B p40 and IL-1β and the restoration of the levels of anti-inflammatory mediators IL-10, IL-4, and TGF-β [109]. In line with these results, Carvajal et al. [85] showed that in animals fed a methionine choline-deficient diet, these nanoparticles not only decreased lipid peroxidation but also attenuated liver inflammatory markers such as C-C Motif Chemokine Ligand 5 (CCL5) and Il-1β, as well as diminishing the proportion of proinflammatory fatty acids. Interestingly, cultured 3D Hep G2 cells challenged with a mixture of palmitic and oleic acid to resemble lipid-induced inflammation in humans showed a significant reduction in the release of proinflammatory cytokines, such as TNF-α and IL-8, when treated with a complex of zinc salt of mefenamic acid, hydroxypropyl-βcyclodextrin, and CeO_2_NPs [109].

During the development of chronic liver disease, hepatocytes can eventually transform to a malignant phenotype and lead to the development of HCC, an inflammation-induced cancer. Adebayo et al. [110] showed that prophylaxis with CeO_2_NPs yielded a reduction in iNos and COX-2 expression in the liver of mice administered with diethylnitrosamine (DEN). In addition, rats with chronically DEN-induced HCC and treated with nanoceria presented reduced macrophage infiltration and M2 marker gene expression (Il-1β, TNF-α, iNOS, and COX-2). The hepatic phosphoproteomic analysis of these animals revealed that the nanoparticles also altered the phosphorylation of genes related to cell–cell and cell–matrix adhesion [111]. 

Significant complications due to end-stage chronic liver disease eventually lead to liver surgery or transplantation. The restoration of blood flow to a previously ischemic liver post-surgery leads to an exacerbation of cellular dysfunction and death [112]. Prophylactic treatment with nanoceria decreased hepatic ischemia reperfusion injury cell death by attenuating the levels of the inflammatory mediators myoglobin, macrophage derived chemokine, plasminogen activator inhibitor 1, macrophage inflammatory protein 2, and Von Willebrand factor [113]. 

Liver dysfunction secondary to other diseases could also potentially be treated with CeO_2_NPs. Sepsis is the most common cause of mortality in intensive care units and one of its more serious complications is liver dysfunction, which leads to disease progression and death [114]. LPS-induced sepsis resulted in high animal mortality, systemic inflammation and liver damage. Increased survival in rats treated with CeO_2_NPs was associated with a decreased serum inflammation. In the liver, nanoceria also ameliorated LPS-induced morphology distortion and diminished the protein expression of iNos, Hmgb1, and MyD88, as well as the phosphorylation of p38 MAPKp44/42-MAPK [115]. In agreement with these results, Hashlem et al. [83] found that CeO_2_NPs protected against liver injury in D-GALN/LPS-induced hepatotoxicity. Treatment with the nanoparticles reduced lipid peroxidation and iNos expression while augmenting cytosolic Nrf2 and, as a consequence, reducing heme oxygenase 1 (HO-1).

In summary, a compelling amount of data strongly indicate that CO_2_NPs behave as powerful anti-inflammatory agents. However, further studies are necessary to accurately define the signaling pathways accounting for this phenomenon. 

## 5. Hepatocellular Carcinoma

Primary liver cancer includes HCC, cholangiocarcinoma, and other types of liver cancer. Among primary liver cancers, HCC is the most frequent histological subtype in approximately 70% to 80% of cases [116]. Patients with liver cancer are often asymptomatic in early stages and do not present with typical liver symptoms, such as jaundice, liver failure, and ascites, until they progress to advanced stages.

Globally, HCC is the fifth most common cancer type and the third leading cause of cancer-related death worldwide [117], with more than one million cases diagnosed each year around the world [117]. HCC is a tumor associated with chronic inflammation and fibrosis arising from different etiologies, including hepatitis B and C and alcoholic and nonalcoholic fatty liver diseases [118,119]. In Western countries, NAFLD is one of the most common liver diseases that promotes the development of HCC [120]. 

### 5.1. Oxidative Stress and Inflammation Mediate HCC Development

Oxidative stress has a key pathological role contributing to the initiation and progression of HCC [121]. When the redox equilibrium is disrupted, either by increased ROS production and/or due to an insufficient response of natural defense systems, key cellular processes such as proliferation and apoptosis are modified [18,122].

Regardless of their etiopathogenic origin and the different molecular mechanisms inherent to each etiology, chronic liver inflammation and the resulting cirrhotic microenvironment are the main factors involved in the onset and progression of HCC [118]. About 80% to 90% of HCC cases originate from cirrhosis caused by chronic inflammation of the liver [123], with death of epithelial cells being the main trigger of the inflammation associated with hepatocarcinogenesis. The pathways which contribute to inflammation-mediated hepatocarcinogenesis include cytokine signaling (TNF-α, IL-6, NF-κB, JNK, STAT3), innate immune signaling, and adaptive immunity [118].

### 5.2. Current Therapeutic Approaches in HCC

Cancer is associated with a poor prognosis. First-line treatment methods in the management of HCC include surgical interventions, cytotoxic chemotherapies and radiation, liver transplantation, microwave ablation, percutaneous ethanol injection, radiofrequency ablation, radiation therapy, supportive care, surgical resection, transcatheter arterial chemoembolization, high-intensity focused ultrasound ablation, percutaneous acetic acid injection, percutaneous cryosurgery, sorafenib, transcatheter arterial chemotherapy, transarterial radioembolization, intra-arterial infusion chemotherapy, systemic chemotherapy, portal vein chemotherapy, portal vein embolization, and their combinations [124]. Conventional therapies can be ineffective and the coexistence of cirrhosis and HCC in the same patient complicates possible therapeutic strategies. The current systemic treatments of HCC are based on molecular targeted therapies. Sorafenib, lenvatinib, cabozatinib, or regorafenib, as well as the antiangiogenic antibody ramucirumab, are considered effective therapies in patients with advanced HCC [125]. However, clinical trials have found only a modest improvement in survival, and overall the median survival continues to be approximately 1 year [126]. Thus, although the new drugs available improve clinical outcomes, the still insufficient effects on disease progression and the emergence of resistance episodes reveal the need to develop new therapies for HCC [127,128].

In this scenario, the development of novel antioxidants able to circumvent the limitation of the classical antioxidants is a logical therapeutic approach. Antioxidants have been described as substances that delay, prevent, or remove oxidative damage to a target. Many antioxidant compounds, enzymes, and nitric oxide inhibitors have been studied for treating chronic inflammation and cancer, some of which have also been evaluated in clinical trials. However, the results to date are suboptimal, mainly due to their low systemic bioavailability and insufficient levels at the target sites.

Previous studies in animals and in liver cancer cells demonstrated that antioxidants are treated as one of the promising strategies to prevent liver cancer [129]. Furthermore, it has been reported that the combination of certain chemotherapeutic drugs and antioxidants could reduce drug resistance, sensitizing the liver cancer cells to chemotherapeutics and thereby improving the efficacy of anti-cancer therapy [130].

### 5.3. CeO_2_NPs as a New Therapeutic Tool in HCC

Nanotechnology has achieved relevance in biomedical research and nanomedicine has emerged as a new treatment option for tumor therapy [131,132]. Among various nanoparticles, CeO_2_NPs have shown promise in a number of applications [133,134].

To act as therapeutic agents, CeO_2_NPs must have a large surface area and reactivity, as well as a wide biocompatibility without systemic toxicity for normal cells and tissues. Different studies have shown that CeO_2_NPs can be toxic to cancer cells by increasing the level of ROS or by targeting the nuclei of tumor cells without affecting the surrounding normal tissue [135,136]. CeO_2_NPs have also been reported to have anti-invasive properties and the ability to sensitize cancer cells to radiation therapy and chemotherapy [137,138,139]. On the other hand, it has been reported that CeO_2_NPs could prevent metastasis and inhibit apoptosis by repressing the ASK1-P38/JNK-NF-κB signaling pathway [138]. 

**Figure 4:**
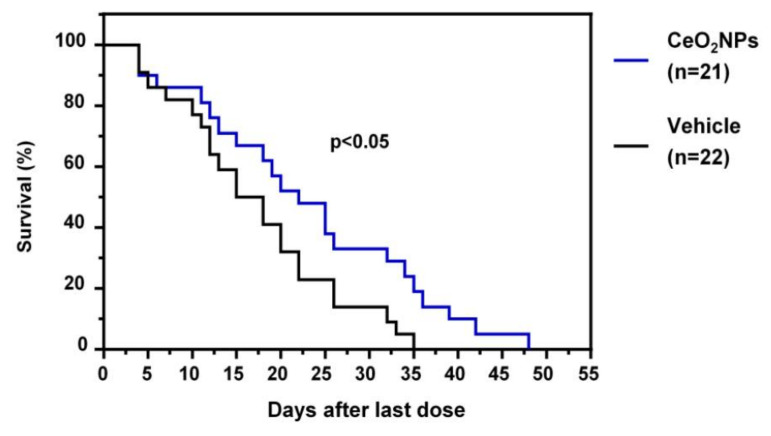
Effect of CeO_2_NPs on survival. HCC rats randomly received two weekly doses of CeO_2_NPs or vehicle through the tail vein at weeks 16 and 17, and their survival was analyzed (adapted from reference [111]).

More recently, Fernández-Varo et al. [111] considered that CeO_2_NPs could be an nanoparticle-based therapy platform in HCC. HCC was induced in rats by the i.p. chronic administration of DEN for 16 weeks. Rats with HCC were treated with CeO_2_NPs i.v. at weeks 16 and 17. The analysis of tissue distribution showed that nanoceria was mainly accumulated in the liver and significantly decreased hepatic macrophage infiltration and reduced the inflammatory M1 gene expression profile, such as IL1β, TNFα, IL6, iNOS, and COX-2. Nanoceria treatment increased liver apoptotic activity, while cell proliferation was attenuated. The authors also investigated the effects of CeO_2_NPs on kinase-driven signaling pathways using mass spectrometry. Phosphoproteomic analysis revealed that CeO_2_NPs affected the phosphorylation of proteins mainly related to cell adhesion and RNA splicing. The analysis of the effect of CeO_2_NPs on hepatic lipid metabolism showed decreased phosphatidylcholine-derived arachidonic acid and a reversal in the HCC-induced increase in linoleic acid in several lipid components. Furthermore, CeO_2_NPs decreased the serum alpha-protein levels and improved the survival of HCC rats (Figure 4). The effect of CeO_2_NPs on overall survival was similar to that observed with sorafenib, which indicates that these nanoparticles are at least as effective as sorafenib under the conditions studied. On the other hand, the intracellular uptake of CeO_2_NPs by human ex vivo perfused livers and human hepatocytes was analyzed. The results obtained demonstrated nanoceria uptake by ex vivo perfused human livers and in vitro human hepatocytes. These results indicate that the antioxidant properties of CeO_2_NPs partially revert the cell mechanisms involved in tumor progression and significantly increase survival in HCC rats. These findings suggest that CeO_2_NPs alone, or in combination with the current molecular targeted therapies, could be effective in stopping or attenuating tumoral progression in patients with HCC [111].

Despite these promising biomedical applications, most of the CeO_2_NPs used in these previous studies were naked or weakly protected by surfactants. This circumstance causes the appearance of many difficulties in in vivo practice, such as the aggregation and elimination of particles by the mononuclear phagocyte system. This situation could cause a decrease in activity and a shorter circulation time of the nanoparticles. To avoid this situation, hydrophilic polymers such as polyethylene glycol (PEG) have been used in an attempt to construct surface coatings of CeO_2_NPs with a better nanoparticle stability and modified surface charges. PEG is considered to be the most effective polymer for improving biocompatibility and adapting the surface charge of inorganic nanoparticles [140]. In this sense, alendronate was found to be an ideal anchor for inserting PEG, specifically PEG600, on the surface of CeO_2_NPs and obtaining improved nanoparticle stability and reduced cytotoxicity in normal human liver cells [141].

## 6. Conclusions

Although our knowledge of the biological protective properties of CeO_2_NPs against free radicals dates back less than two decades, and that the first studies in the field of hepatology are less than one decade old, there is abundant evidence showing the therapeutic potential of this nanomaterial in liver diseases. In fact, oxidative stress is considered a key pathogenic mechanism contributing to the initiation and progression of most liver diseases and, therefore, strategies aimed at reducing free radicals are of great interest. In contrast to classical antioxidants, which have short and no targeted activity, CeO_2_NPs present multienzimatic activity, high liver tropism, long residence time in liver tissue, and lack of consumption during the redox reactions, which significantly increase their potential therapeutic activity in liver diseases. In agreement with this, protective effects have been found in different experimental models of liver disease, including liver fibrosis, steatohepatitis, hepatocellular carcinoma and liver regeneration. Moreover, studies generally do not show toxicity under standard therapeutic doses. Nonetheless, some aspects have yet to be clarified. Thus, although the mechanisms of action are beginning to be characterized, they still need to be completely elucidated and understood. Future work also includes characterization of the evolution of nanomaterial in different in vivo scenarios, the knowledge of their cellular and subcellular distribution, and possibilities of controlled bioditribution and functionalization. Overall, current evidence places CeO2NPs as a simple and powerful therapeutic approach for highly prevalent liver diseases.

## Abbreviations

Acetaminophen	APAP
adenosine triphosphate	ATP
antioxidant response element	ARE
augmenter of liver regeneration	ALR
carbon tetrachloride	CCl_4_
cerium (IV) oxide	CeO_2_
cerium (III) oxide	Ce_2_O_3_
cerium oxide nanoparticles	CeO2NPs
cyclooxygenase 2	Cox-2
damage associated molecular patterns	DAMPs
desferoxamine	DES
diethylnitrosamine	DEN
extracellular matrix	ECM
fatty acid synthase	FASN
glutamate-cysteine ligase, catalytic subunit	GCLC
glutathione	GSH
glutathione reductase	GR
glutathione-S-transferase	GST
hepatic stellate cells	HSCs
hepatocyte growth factor	HGF,
hepatocellular carcinoma	HCC
heme oxygenase 1	HO-1
IL-6 receptor	IL-6R
inducible nitric oxide synthase	iNOS
interleukin 1 alpha	IL-1α
interleukin 6	IL-6
intraperitoneally	i.p.
intravenously	i.v.
IkB kinase	IKK
Janus xineses	JAK
knockout	KO
malondialdehyde	MDA
methionine choline deficient	MCD
mitogen-activated protein kinase	MAPK
metabolic dysfunction-associated fatty liver disease	MAFLD
myeloperoxidase	MPO
NADPH quinine oxidoreductase 1	NQO1
N-acetyl cysteine	NAC
nicotinamide adenine dinucleotide phosphate	NADPH
NF-E2-related factor 2	Nrf2
neutrophil cytosol factor 2	Ncf2
nitric oxide	NO
non-alcoholic fatty liver disease	NAFLD
non-alcoholic steatohepatitis	NASH
nuclear factor	NF
nuclear factor kappa-light-chain enhancer of activated β cells	NF-κB
partial hepatectomy	PHx
polyethylene glycol	PEG
prostaglandin-endoperoxide synthase 1	PTGS1
reactive oxygen species	ROS
reactive nitrogen species	RNS
S6 kinase signal-transduction pathways	TOR
suppressor of cytokine signaling-3	SOCS-3
superoxide dismutase	SOD
tempol	TEM
thiobarbituric acid reactive substances	TBARS
transforming growth factor alpha	TGFα
tumor necrosis factor alpha	TNFα
urokinase-type plasminogen activator	uPA
vascular endothelial growth factor	VEGF
